# Expanded Indications for Hybrid Spinal Fixation Systems; Combined Percutaneous Pedicle Screw Fixation and Open Approaches

**DOI:** 10.3390/jpm16070387

**Published:** 2026-07-20

**Authors:** Thomas Repantis, Ioanna Lianou, Ioannis Papaioannou, Maria Papathanasiou, Lexi de Jager, Andreas Filippopoulos, Andreas Baikousis

**Affiliations:** Orthopedic Department, General Hospital of Patras, 26224 Patras, Greece; jlianou@med.uoa.gr (I.L.); john-pane1984@hotmail.com (I.P.); mpapath@outlook.com (M.P.); lexidejager@hotmail.com (L.d.J.); andreas.filippopoulos@hotmail.com (A.F.); agbaikousis@gmail.com (A.B.)

**Keywords:** minimal spine surgery, percutaneous, screw fixation, hybrid methods, min-open

## Abstract

**Background/Objectives**: Minimally invasive (percutaneous) pedicle screw fixation (PPSF) was initially introduced for the treatment of degenerative spinal deformities. Since then, its indications have progressively expanded to a broad spectrum of spinal pathologies. This method has gained increasing acceptance in spinal surgery due to lower morbidity when compared with conventional open procedures. This study presents a comprehensive review of the recent literature on hybrid minimally invasive spinal instrumentation techniques, focusing on the combined use of PPSF with open or mini-open approaches and their roles in personalized surgical management. **Methods**: A literature search was conducted in PubMed and Web of Science to identify studies reporting expanded indications of percutaneous pedicle screw fixation (combined with other approaches), novel surgical techniques, and their clinical outcomes. **Results**: Thirty-five studies met the inclusion criteria and were categorized according to pathology. Most included studies were retrospective observational investigations corresponding to Oxford CEBM Levels III–IV evidence, with a smaller number of prospective studies and systematic reviews. **Conclusions**: The findings from this review highlight the expanding role of hybrid methods in the management of complex spinal disorders. These approaches provide adequate stability and enable decompression or deformity correction, while minimizing tissue trauma, blood loss, and perioperative morbidity, thereby facilitating improved recovery and functional outcomes. The included literature predominantly represents moderate levels of evidence, supporting a patient-specific, pathology-driven surgical strategy that optimizes individualized outcomes in spinal surgery.

## 1. Introduction

Modern spinal surgery has evolved from fundamental principles described by ancient Egyptian and Greek scholars [1]. Conventional open spine surgery has long been the standard of care for spinal disorders, despite substantial limitations, including increased blood loss and significant and often irreversible paraspinal muscle injury resulting from muscle retraction and soft-tissue dissection. This can lead to delayed rehabilitation and a delayed return to daily activities [2]. In parallel with advances across surgical disciplines in favor of minimal soft tissue disruption, which emphasizes “leaving the smallest footprint”, spine surgery has progressively shifted toward minimally invasive options [3]. Minimally invasive spine surgery (MISS) has been widely adopted to overcome morbidity related to open approaches. MISS initially gained popularity in the treatment of degenerative spine disease and later expanded to more complex spinal procedures. Specifically, the evolution of MISS began with the introduction of the Yaşargil microscope in 1967, which laid the foundation for minimally invasive spinal procedures. Further advances were made in the following decades, most notably with Magerl’s application of percutaneous transpedicular external fixation of the lumbar spine in the 1980s [4]. Similarly, Leu et al. reported the successful use of dorsolateral percutaneous interbody fusion in 1993 [5]. Over time, the indications for minimally invasive spinal fusion expanded to include more complex spinal conditions, such as degenerative spine disease, spinal oncology, deformities and trauma [6]. Clinical outcomes following MISS have been shown to be comparable with those from conventional open procedures, while recovery time and pain tend to be reduced [7].

Despite these favorable outcomes, MISS is inherently restricted by limited surgical exposure and restricted access to anatomical structures, possibly compromising the completeness of management of spinal pathologies [8,9,10]. The extent of spinal involvement and the severity of the segmental damage in most cases are influenced by patient age [11], thus supporting the selective use of MISS, even in complex cases, as percutaneous instrumentation can minimize morbidity and surgery-related risks [2]. The use of minimally invasive procedures necessitates intraoperative imaging and involves a steep learning curve, requiring advanced surgical skills and specialized training, and it may result in increased radiation exposure for both the surgical team and the patient [12,13]. Interestingly, robot-guided screw placement is associated with a low to almost negligible learning curve [14]. Given the versatility of the percutaneous pedicle screw technique, this method can be combined with mini-open procedures as an optimal alternative to traditional open approaches [15]. Such hybrid methods have demonstrated comparable clinical outcomes, particularly in patients for whom extensive open treatment can pose increased risk due to a compromised general health status [16]. The aim of this study is to provide a comprehensive review of the literature on the expanded application of percutaneous pedicle screw fixation in combination with open or mini-open approaches. The indications for this technique in complex and/or multilevel spinal pathologies, as well as the associated clinical outcomes, are also analyzed, with particular emphasis on its role in patient-specific and personalized surgical decision-making.

## 2. Materials and Methods

This study is a structured narrative review of the literature. The aim of this review is to investigate the expanding use of hybrid spinal fixation systems, specifically percutaneous pedicle screw fixation (PPSF) combined with open and/or mini-open approaches, with an emphasis on expanded indications and associated clinical outcomes.

A structured literature search was performed in two electronic databases with wide coverage of orthopedic and spine surgery literature: PubMed (1947 to present) and Web of Science (1900 to present), on 15 March 2026. The literature search was restricted to these databases due to their extensive coverage of biomedical and surgical research. The search strategy combined the following terms: (“percutaneous pedicle screw” OR “minimally invasive pedicle screw” OR PPSF) AND (“hybrid surgery” OR “combined approach” OR “mini-open” OR “open decompression” OR “open fusion”) AND (“spinal disorders” OR “spine trauma” OR “spinal deformity” OR “spondylodiscitis” OR “spinal metastasis”). The search strategy was adapted for each database.

**Figure 1:**
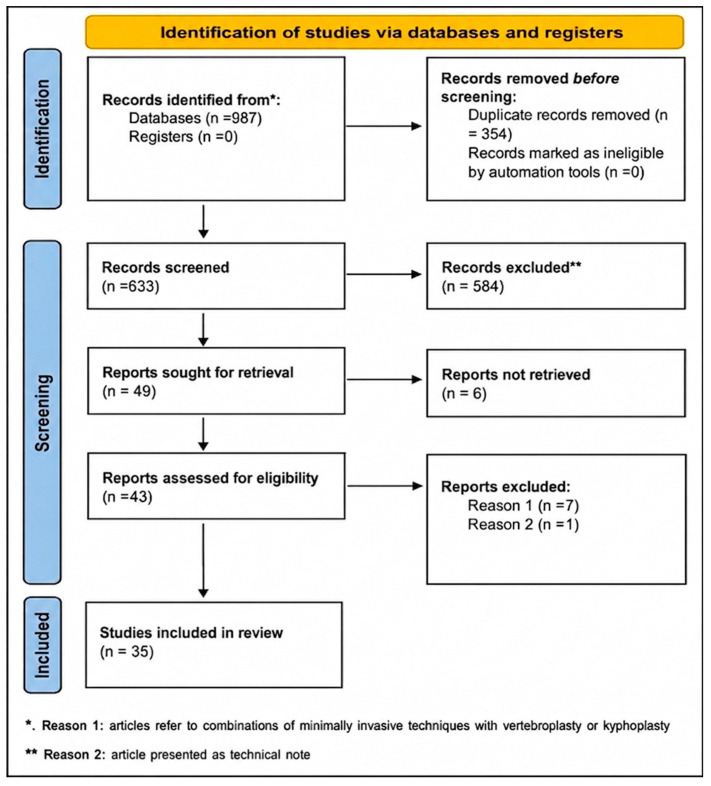
PRISMA 2020-guided flow diagram illustrating study identification and selection process. * Records identified in both databases (Pubmed and Web of Science). ** Records excluded by the authors without automation tools.

To enhance the transparency and reproducibility of study identification and selection, a PRISMA 2020-guided flow diagram was used to document the search process (Figure 1). This approach was used to report the selection pathway and does not indicate that the present study represents a formal systematic review. Due to the substantial heterogeneity in study designs, patient populations, surgical techniques, and reported outcomes, a quantitative synthesis or meta-analysis was not performed.

Eligible studies included full-text English-language publications reporting the use of PPSF combined with open or mini-open spinal procedures. Study designs included case reports, case series, observational studies, comparative studies, systematic reviews, and meta-analyses. Studies involving single-stage or staged hybrid procedures across any spinal region were included. No publication date restrictions were applied. Exclusion criteria included technical notes, short communications, expert opinions, and letters to the editor, as well as studies lacking sufficient detail regarding the surgical technique or pathology. Studies involving only minimally invasive combinations without an open component (e.g., PPSF with vertebroplasty or kyphoplasty) were excluded. Non-English publications were not included.

The review was conducted in accordance with the SANRA (Scale for the Assessment of Narrative Review Articles) guidelines to ensure methodological transparency and structured reporting. Given the narrative nature of this review and the heterogeneity of the included study designs, formal risk-of-bias assessment tools were not applied. Instead, studies were categorized according to the Oxford CEBM Levels of Evidence to provide an overview of the evidence strength. The limitation of restricting the search to PubMed and Web of Science was acknowledged.

Although a PRISMA-guided reporting framework was used to improve transparency, the present study should be considered a structured narrative review rather than a formal systematic review because no protocol registration, risk-of-bias assessment, or quantitative synthesis was performed. A formal risk-of-bias assessment was not performed because of the narrative design and the substantial heterogeneity of the included studies.

## 3. Results

### 3.1. Included Studies

A total of 35 studies met the inclusion criteria and were categorized according to pathology: nine studies on degenerative disease and spinal deformity, 11 on traumatic spinal pathology, six on infectious spinal disease, six on metastatic spinal disease, and three review articles/meta-analyses. (Table 1, Table 2, Table 3, Table 4 and Table 5) [16,17,18,19,20,21,22,23,24,25,26,27,28,29,30,31,32,33,34,35,36,37,38,39,40,41,42,43,44,45,46,47,48,49,50]. Five case reports described the application of this approach in specific clinical scenarios [18,19,20,24,29].

Of the 35 included studies, 23 were retrospective studies, four were prospective studies (including two comparative prospective studies), five were case reports, and three were review articles/meta-analyses. Most of the evidence corresponded to Oxford CEBM Levels III–IV.

### 3.2. Quality Assessment

Overall, the included studies represented a range of evidence levels (I–V, Oxford CEBM), reflecting predominantly moderate-quality and some high-quality (Level I evidence) data that support the evolving use of hybrid percutaneous and open spinal fixation techniques. This grading highlights both the growing clinical acceptance of hybrid techniques and the need for further prospective controlled studies to establish definitive comparative effectiveness.

## 4. Discussion

### 4.1. Applications of PPSF Combined with Open Procedures in Degenerative Spine Pathologies and Deformities

In recent years, various minimally invasive spinal surgery methods have emerged as promising alternatives to traditional open procedures. Even in complex spinal pathologies, MISS methods, including PPSF, can be combined with open approaches to expand the indications for PPSF. This review consolidates evidence from a range of studies assessing different interventions for spinal pathologies (i.e., degenerative diseases, spondylolisthesis or deformities) focusing on the effectiveness of hybrid approaches. These procedures can be performed either in one stage or in a two-stage procedure (Table 6).

The studies reviewed highlight the growing body of evidence supporting the efficacy of a hybrid approach, achieved by combining minimally invasive lumbar interbody fusion (mini-ALIF) with percutaneous screw fixation. Lee et al. (2004) demonstrated that combining mini-ALIF with PPSF, which were performed in the same surgical procedure, led to important clinical improvements [17]. These included less muscle injury, no epidural scar formation, and earlier discharge when applied in patients with isthmic spondylolisthesis accompanied by leg pain [17]. This is consistent with the findings of other studies, for example, Anderson et al. (2011) who revealed high fusion rates and significant improvements in pain scores following ALIF combined with rhBMP-2 and allograft, performed under the same general anesthetic, in patients with degenerative lumbar diseases [28]. Both studies emphasize that minimally invasive approaches, when combined with different methods of spine surgery, may reduce surgical trauma and improve recovery times, supporting their growing role in spinal surgery. Similarly, hybrid techniques combining percutaneous pedicle screw fixation with posterior lumbar interbody fusion (PLIF) and central decompression, as reported by Kim et al. (2011) resulted in satisfactory outcomes [39]. Demonstrated outcomes include less iatrogenic muscle injury or muscular denervation and reduced blood loss compared to traditional methods. This technique seems to be particularly beneficial for patients with multilevel spondylolisthesis or stenosis, where conventional open surgery may lead to more significant morbidity [39]. A hybrid method consisting of minimally invasive decompression and PLIF was demonstrated by Kotani et al. (2012) [45], whose study contributed valuable insight into the superiority of minimally invasive posterior lumbar fusion (MIS-PLF) over open posterolateral fusion for the efficient treatment of degenerative spondylolisthesis with spinal stenosis. According to the results from this comparative study, the MIS-PLF group demonstrated better mid-term results concerning pain and function, with a significantly lower complication rate. In terms of surgical time, both groups demonstrated equivalent results, which are related to the learning curve with PPSF and bone grafting in a small surgical plane. Moreover, Barbagallo et al. (2014) presented results from the application of a mini-open transforaminal interbody fusion combined with PPSF technique in patients with multi-level degenerative diseases [46]. Their findings support the role of minimally invasive methods combined with open or mini-open approaches as safe and efficient alternatives with excellent clinical outcomes. No reported neurological deficits related to pedicle screw placement or interbody cages were described [46]. Similarly, studies by Ulutaş et al. (2015) and Wang and Bordon (2016) confirmed that minimally invasive techniques can offer comparable, and in some cases superior, results to open procedures in terms of complication rates, recovery time and clinical outcomes [47,48]. In particular, Wang and Bordon presented results post-treatment of patients with severe spinal deformities through a method combining mini-open pedicle subtraction osteotomies and PPSF.

An interesting approach was reported by Heo et al. (2019) who compared the outcomes of PPSF with a reduction system combined with mini-open decompression and posterior lumbar interbody fusion (PLIF) versus a conventional open approach in the treatment of lumbar spondylolisthesis [49]. Their research suggested that PPSF with PLIF provided better maintenance of lumbar lordosis and the segmental angle compared to the traditional open methods, resulting in superior clinical and radiological outcomes. This reinforces the notion that hybrid methods may improve both immediate and long-term surgical results, even in complex spinal deformities. Moreover, Liu et al. 2020 demonstrated the efficacy of percutaneous pedicle screw fixation combined with a Schwab grade 4 osteotomy in posttraumatic thoracolumbar kyphosis [50]. The study reported comparable outcomes to open procedures, with less blood loss and reduced lower back pain postoperatively [50]. Hybrid minimally invasive approaches combining PPSF with fusion or decompression techniques consistently offer significant benefits in degenerative spine disease and deformity management. These advantages are evidenced by reduced surgical trauma, faster recovery and favorable long-term outcomes.

### 4.2. Applications of Hybrid Methods on Trauma Cases or Post-Traumatic Deformities

Various studies highlight the success of hybrid methods, such as PPSF combined with endoscopic decompression or open surgical approaches in the management of complex spinal fractures or post-traumatic pathologies. Studies by Park et al. (2018) Huang et al. (2020) and Bai et al. (2025) [21,24,27] underscore the effectiveness of minimally invasive approaches in managing thoracolumbar burst fractures with severe spinal stenosis. These hybrid methods, combining PPSF with microscopic decompression, yield excellent results with reduced blood loss and better pain management. This in turn leads to quicker recovery times, while avoiding serious complications associated with traditional open surgeries. Interestingly, a hybrid method of short-segment PPSF with a small laminectomy can effectively correct the deformity (kyphotic) and restore the anatomy of the affected vertebra in patients with thoracolumbar burst fractures and symptomatic spinal compression [21]. Moreover, Eck. (2011) introduced a minimally invasive technique for treating an L3 burst fracture using a combination of a one-stage anterior corpectomy and L2–L4 interbody fusion via a direct lateral approach, combined with PPSF [18]. This approach resulted in clinical and neurological improvements with less postoperative morbidity and blood loss. This case highlights the advantages of minimally invasive techniques, including smaller incisions, reduced anesthesia burden, and faster recovery, which are particularly relevant in patients with comorbidities.

PPSF combined with a posterior open approach has been used for the treatment of multilevel non-contiguous spinal fractures [43]. Sebastian et al. (2015) presented a hybrid method combining open occiput to T3 posterior fusion with percutaneous posterior instrumentation for C1 ring fractures, C7–T1 extension fractures, and T9–T10 extension fractures in a patient with spinal ankylosis [19]. This technique proved effective for multiple spinal injuries, especially in patients with concomitant comorbidities, due to reduced perioperative morbidity and blood loss. Moreover, percutaneous short-segment pedicle screw fixation combined with mini-decompression for non-contiguous lumbar burst fractures, as reported by Kim et al. (2018), provides a minimally invasive non-fusion method that preserves motion, as well as achieving low morbidity and excellent functional outcomes. This technique is particularly useful in young patients with neurological deficits or multiple fractures [20].

Todeschi et al. (2021) compared minimally invasive techniques with open posterior fusion in A3 and A4 type thoracolumbar fractures [25]. These findings indicated that a staged hybrid method, including PPSF followed by delayed stage mini-open anterolateral corpectomy and interbody fusion, resulted in superior long-term clinical and radiological outcomes. Additionally better maintenance of spinal alignment was found when compared with open surgery procedures. This method also allows for safe removal of screws and the release of vertebral movements. Hybrid methods were also utilized in rare cases, such as those described by Zhang et al. (2022) and Bravo et al. (2025), including double non-contiguous fractures with traumatic spinal stenosis fractures or acute traumatic thoracic spondyloptosis [26,29]. Bravo et al. reported a rare case of posterior T9 spondyloptosis, treated with PPSF, T9 vertebrectomy and T9–T10 decompression [29]. These cases demonstrated that hybrid methods offer effective stabilization and allow for better spinal function recovery, while minimizing the risk of complications and reducing the need for extensive open procedures.

In conclusion, the studies reviewed here highlight the promising role of minimally invasive and hybrid techniques in the management of traumatic spinal fractures. These methods offer significant advantages over traditional open procedures by providing faster recovery, reduced morbidity, and better long-term outcomes. The ability to tailor surgical approaches to the patients’ needs, based on the severity and location of the fractures, is significant to optimizing results.

### 4.3. Application in Infectious Spinal Pathologies

The adoption of minimally invasive spine surgery for the treatment of spinal infections, including tuberculous and pyogenic spondylodiscitis, has shown promising results. This review synthesizes evidence from various studies exploring the efficacy and safety of minimally invasive techniques, encompassing hybrid methods of percutaneous pedicle screw fixation combined with anterior and posterior open procedures. Kandwal et al. (2012) compared two different approaches for tuberculous spondylodiscitis [30]. The first group was treated with video-assisted thoracoscopic surgery combined with anterior debridement and fusion and the second group was treated with percutaneous pedicle screw fixation, mini-open decompression and fusion [30]. Both approaches demonstrated good fusion rates and functional outcomes with significantly reduced blood loss and shorter operative duration. The correction of kyphosis was better maintained in cases with lesser degrees of deformity. Similarly, Garg and Vohra. 2014, presented the outcomes of MISS combined with open approaches in treating extended vertebral body destruction due to spinal tuberculosis. They performed a hybrid approach that combined posterior minimally invasive spinal transpedicular debridement and percutaneous pedicle screw fixation with anterior ventral column reconstruction. The results demonstrated near-perfect neurological recovery, avoidance of complications, and no progression of deformities, further supporting the efficacy of MISS in complex infectious spinal cases [31].

Lin et al. 2014 conducted a retrospective study comparing the results of a two-stage procedure combining anterolateral interbody fusion with either PPSF (MISS group) or conventional posterior open surgery for the treatment of pyogenic spondylodiscitis [32]. The MISS group exhibited superior postoperative pain control, reduced intraoperative blood loss, and shorter operative time compared to the open approach. Long-term neurological outcomes were similar between both groups, and there was no infection recurrence, suggesting that the MISS approach is a viable option for treating pyogenic spondylodiscitis with comparable long-term results to traditional open surgery [32]. In another study, Lin et al. (2015) investigated the safety and efficacy of a hybrid method involving mini-open anterior debridement and lumbar interbody fusion combined with PPSF, through a modified anterolateral interbody fusion (ALIF) for single-level lumbar pyogenic spondylodiscitis [33]. This hybrid approach proved to be a safe alternative to conventional methods, with fewer postoperative complications, less surgical site trauma, and minimal blood loss. Therefore, this highlights the benefits of hybrid methods including posterior percutaneous instrumentation in managing infections of the lumbar spine [33]. Similarly, Wang et al. (2017) assessed the outcomes of a novel one-stage procedure combining extreme lateral interbody fusion (XLIF) and PPSF for the treatment of lumbar spine tuberculosis [34]. This hybrid method resulted in shorter hospitalization, the retention of spinal stability, faster recovery, less blood loss, and a lower rate of infection and complications, particularly among older patients. These findings suggest that this combined approach is especially beneficial for patients who may be at higher risk for complications, such as the elderly [34]. The treatment of thoracolumbar spondylodiscitis was evaluated by Zhang et al. (2020) who reported initial outcomes from the application of a hybrid technique combining PPSF and a mini-open approach to debride the affected disc-bone space and perform decompression [35]. Their study demonstrated that hybrid methods can be both effective and safe, offering favorable outcomes such as reduced blood loss, shorter surgical duration, and improved postoperative pain management. These findings further support findings from the known literature, which demonstrate the role of minimally invasive techniques combined with open approaches in the treatment of thoracolumbar infections, where managing perioperative trauma is crucial to improving patient recovery [42].

### 4.4. Applications of Hybrid Method in Metastatic Spine Disease

Findings from the known literature highlight the evolving role of minimally invasive spine surgery (MISS) combined with open procedures in the management of metastatic spinal disease. Hybrid methods involving percutaneous pedicle screw fixation (PPSF) combined with or without limited decompression demonstrate outcomes comparable to conventional open surgery, with several additional perioperative advantages. A consistent outcome among the included studies is the effectiveness of these methods in achieving adequate pain control and neurological recovery. In particular, Lin et al. (2013) reported that long-segmental posterior minimally invasive fixation combined with decompression is both safe and effective in improving pain and neurological outcomes in patients with symptomatic spinal metastases [36]. Similarly, Hamad et al. (2017) demonstrated that PPSF, either alone or in combination with mini decompression, maintains or improves functional outcomes in most patients [41]. These findings support the concept that less invasive methods of instrumentation combined with mini-open procedures can adequately address spinal cord compression and instability in selected patients. Comparative studies from Kumar et al. (2017) [38] and Miscusi et al. (2015) [40] showed similar outcomes between hybrid methods, including PSSF and open procedures in terms of pain relief, functional status, and neurological recovery. Importantly, MISS approaches were associated with reduced intraoperative blood loss, shorter hospital stays, decreased opioid consumption, as well as earlier initiation of adjuvant therapies such as chemotherapy and radiotherapy [38,40]. These factors are particularly relevant in oncologic patients, where minimizing surgical morbidity and facilitating rapid recovery are critical for overall prognosis and quality of life.

Interestingly, Rao et al. (2014) proposed a treatment algorithm based on patients’ life expectancy, suggesting that the extent of surgical intervention should be tailored accordingly [37]. Patients with limited survival may benefit from less invasive decompression and stabilization, while those with longer survival (longer than 12 months) may still require more extensive procedures. This may include local or marginal tumor resection, open decompression, vertebral body reconstruction, and multilevel stabilization [37]. This aligns with the principles of personalized medicine, emphasizing individualized treatment planning based on patient prognosis, disease burden, and functional status. Moreover, the complication rate reported across studies favors the application of hybrid methods, combining percutaneous and open approaches. Colangeli et al. (2020) revealed lower morbidity and shorter hospital stays for patients treated for spinal metastases, while maintaining similar efficacy in pain and neurological outcomes [16]. The reduced complication rates, including lower infection risk and decreased need for transfusion, further support the adoption of minimally invasive techniques in appropriately selected patients [16]. These findings underscore the value of hybrid minimally invasive approaches for the personalized management of metastatic spinal disease, optimizing both efficacy and patient recovery.

### 4.5. Limitations of Minimally Invasive Spine Surgery (MISS) and Potential Benefits of Robot-Assisted and Image-Guided Navigation Systems

The expansion of minimally invasive spine surgery has led to increased use of image-guided techniques, including two-dimensional fluoroscopy, which exposes both surgeons and patients to radiation levels proportional to the duration of the procedure [12]. Although advances in intraoperative imaging have introduced newer technologies, the literature remains conflicted regarding radiation exposure, with some studies suggesting reduced exposure compared with conventional methods [51]. Several strategies may be implemented to minimize the radiation dose during PPSF. Ultrasound-guided techniques represent a promising and cost-effective imaging modality that may be combined with PPSF to reduce radiation exposure and broaden MISS indications [52]. In addition, high-resolution three-dimensional imaging systems, such as the O-arm and three-dimensional C-arm, have been shown to improve pedicle screw accuracy while reducing radiation exposure and maintaining comparable operative times [53].

PPSF is associated with a steep learning curve and requires advanced surgical expertise. However, robot-assisted pedicle screw placement offers notable advantages over conventional fluoroscopy- or navigation-guided techniques, particularly in terms of accuracy and reduced radiation exposure [14,54]. Although the reported learning curve for robotic systems varies across studies, some evidence suggests a minimal learning curve for robot-assisted screw placement [14]. On the other hand, minimally invasive transforaminal lumbar interbody fusion (MIS-TLIF) has been associated with a steeper learning curve, suggesting that robotic assistance may improve accuracy and reduce pedicle wall violation rates [55].

Among emerging MISS techniques, microscope-assisted and endoscopic decompression methods are gaining increasing acceptance, particularly when combined with other minimally invasive approaches. Unilateral biportal endoscopic decompression combined with PPSF may be applied in complex cases, including spinal infections, tumors, and trauma [54]. Recent evidence suggests that PPSF, by preserving the facet joint capsule and reducing paraspinal muscle injury and fibrosis, in combination with unilateral biportal endoscopic decompression, can achieve satisfactory deformity correction and adequate decompression with favorable short- and mid-term outcomes, even in thoracolumbar fractures with spinal stenosis [27].

Hybrid techniques have demonstrated effectiveness in maintaining sagittal alignment in patients undergoing anterior or lateral lumbar interbody fusion for degenerative disease. However, patient-specific factors—such as multiplanar deformity, coronal imbalance, prior decompression or instrumentation, and elevated body mass index—may still necessitate open surgical approaches [44] (Table 7).

### 4.6. Strengths and Limitations

To the best of our knowledge this study incorporates the most up-to-date literature on the use of percutaneous pedicle screw fixation along with other open or mini-open spine surgical approaches for the treatment of a wide range of spinal diseases and is currently the only review focusing specifically on this promising topic. It synthesizes findings from recent studies, highlighting the evolving and increasingly personalized application of PPSF techniques. However, our analysis has several limitations. The exclusion of additional databases may have limited study capture and is acknowledged as a study limitation. Most of the included studies are retrospective or involve relatively small sample sizes, which may introduce selection bias and limit the generalizability of the results. Additionally, the diversity of surgical techniques, patient populations, and outcome measures makes direct comparison challenging. Long-term outcomes and cost-effectiveness analyses remain underreported and warrant further investigation. Finally, a language bias may be present due to the inclusion of only studies written in English.

## 5. Conclusions

Percutaneous pedicle screw fixation, specifically when combined with mini-open approaches, is a safe and effective alternative to traditional open surgery across various spinal pathologies. Hybrid techniques continually demonstrate reduced perioperative morbidity, facilitate faster recovery, and maintain comparable clinical outcomes, thereby making them especially suitable not only for elderly patients but also for those requiring individualized care. However, the current body of literature is predominantly composed of retrospective and observational studies, limiting the strength of definitive conclusions. Further high-quality studies are warranted to refine indications and standardize outcomes, supporting personalized treatment strategies in spinal surgery, especially in complex cases.

## Figures and Tables

**Table 1:** Hybrid Percutaneous Pedicle Screw Fixation (PPSF) techniques in degenerative spine disease, spondylolisthesis, and spinal stenosis.

Study	Type of Study	Number of Patients	Aim of Study/Spine Pathology	Type of Intervention	Outcomes	Level of Evidence (Oxford CEBM)
Lee et al. 2004 [17]	Retrospective study	73	To evaluate one stage minimally invasive anterior lumbar interbody fusion (mini-ALIF) with posterior percutaneous pedicle screw fixation (PPSF) without decompression; symptomatic spondylolisthesis	Mini-ALIF combined with PPSF	Less muscle injury, no epidural scar or blood transfusion, good pain control and early discharge	III
Anderson et al. 2011 [28]	Retrospective study	50	To estimate anterior lumbar interbody fusion (ALIF) with PPSF; degenerative diseases of lumbar spine	One stage procedure; primary anterior lumbar fusion (ALIF with rhBMP-2 and allograft) and PPSF	Safe method, high fusion rate. Statistically significant results regarding pain scores.	III
Kim et al. 2011 [39]	Retrospective study	42	To describe hybrid surgical method of multilevel PPSF; instability, spondylolisthesis or stenosis.	Foraminal mini decompression with multilevel PPSF	Less iatrogenic muscle injury, postoperative blood loss and back pain	III
Kotani et al. 2012 [45]	Prospective cohort study	80 (43 with minimally invasive lumbar decompression with posterolateral fusion (MIS-PLF) vs. open	To compare outcomes of MIS-PLF with open posterolateral fusion; degenerative spondylolisthesis	PPSF and MIS-PLF vs. open fusion	Better pain/function, lower complication rate (3.8%)	II
Barbagallo et al. 2014 [46]	Clinical series	13	To describe mini transforaminal lumbar interbody fusion (TLIF) with PPSF; multilevel degenerative diseases	Mini open TLIF and PPSF	Safe technique, no neurological deficits or re-operations.	IV
Ulutaş et al. 2015 [47]	Prospective study	35 with MISS and 35 with conventional pedicle screw fixation	To estimate safety and efficiency of MISS; thoracic and lumbar spine degenerative pathologies	Microdiscectomy and cage insertion (TLIF) through midline incision, combined with PPSF	Good sagittal correction, no major complications	II
Wang and Bordon 2016 [48]	Retrospective study	16 patients	To report results from hybrid pedicle subtraction osteotomy and PPSF; coronal and sagittal plane deformities	L2–L3 subtraction osteotomy with PPSF and facet joint or interbody fusion	Reduced soft tissue damage, good alignment	III
Heo et al. 2019 [49]	Comparative study	65 (33 with open transpendicular fixation and 32 with PPSF and posterior lumbar interbody fusion-PLIF)	To study efficiency of PPSF with reduction system in lumbar spondylolisthesis	Open transpendicular fixation and PLIF vs. PPSF with reduction system and PLIF (open laminectomy)	Better lordosis and segmental angle maintenance with PPSF	III
Liu et al. 2020 [50]	Case–control study	34	To present results of the combined Schwab grade 4 osteotomy with PPSF; posttraumatic thoracolumbar kyphosis	Grade 4 osteotomy (egg-shell technique) through mini–open approach combined with PPSF	Less blood loss and low back pain. Similar misplacement rate. No implant loosening, fracture or correction loss reported.	III

**Table 2:** Hybrid Percutaneous Pedicle Screw Fixation (PPSF) in the management of traumatic spine injuries.

Study	Type of Study	Number of Patients	Aim of Study/Spine Pathology	Type of Intervention	Outcomes	Level of Evidence (Oxford CEBM)
Eck. 2011 [18]	Case report	1	To present minimally invasive anterior and posterior fixation; L3 burst fracture	One stage L3 corpectomy with L2–L4 fusion and PPSF	Neurological improvement, less morbidity and blood loss.	IV
Sebastian et al. 2015 [19]	Case report	1	To present a hybrid method for multiple non-contiguous fractures in ankylosis	Open occipitocervical fusion and PPSF T5–L1	Effective in complex trauma	IV
Kim et al. 2018 [20]	Case report	1	To present treatment of non-contiguous burst lumbar spine fractures (L2 and L5 with neurological impairment)	PPSF (short-segment) and posterior mini decompression	Motion preservation, good clinical outcome	IV
Park et al. 2018 [21]	Retrospective study	27	To evaluate PPSF and spinal decompression; single-level burst fracture of thoracolumbar junction (T11–L2) with neurological Deficits	Mini posterior decompression and PPSF	No neurological deterioration, good correction.	III
Ushijima et al. 2018 [22]	Case report	1	To present treatment of non-contiguous fractures of cervicothoracic and thoracolumbar zone in spinal ankylosis and spondylo-epiphyseal dysplasia	Hybrid open + percutaneous fixation	Solid fusion, stable construct	IV
Erichsen et al. 2020 [23]	Retrospective analysis	87 (open vs. PPSF, subgroup of 25 with second stage anterior fusion)	To compare treatment of AOSpine type A3 spines (T11 and L2)	PPSF and thoracoscopic anterior fusion (Mc Cormack Scores ≥ 6 and disk pathology)	Less reduction loss, shorter operating room time	III
Huang et al. 2020 [24]	Case report	1	To present hybrid method of PPSF with transforaminal endoscopic spinal canal decompression; thoracolumbar burst fractures with neurologic deficits	PPSF and transforaminal endoscopic spinal cord decompression (5 patients with persistent neurological deficit)	Safe prosedure, neurologic improvement reported	IV
Todeschi et al. 2021 [25]	Prospective study	110 (66 with PPSF with or without mini-open decompression and staged interbody fusion vs. open instrumentation)	To compare two-stage procedure with PPSF and interbody fusion versus one stage open posterior fusion; thoracolumbar spine fractures (A3 and A4 AOSpine)	PPSF with or without mini-open approach and staged fusion vs. open surgery	Higher fusion rate, better long-term clinical in hybrid group.	II
Zhang et al. 2022 [26]	Retrospective comparative study	64	To compare results from posterior mini-open microscopic decompression and PPSF vs. open treatment; traumatic spinal canal stenosis after AOSpine A3 or A4 fractures	PPSF and mini-open microscopic decompression	Less blood loss, better pain control	III
Bai et al. 2025 [27]	Retrospective study	16	To present PPSF and unilateral biportal endoscopic decompression; thoracolumbar burst fractures with spinal stenosis	Unilateral biportal endoscopic decompression and PPSF	Satisfying deformity correction, good short and mid-term outcomes	III
Bravo et al. 2025 [29]	Case report	1	To present hybrid vertebral shortening method; acute traumatic thoracic spondyloptosis (T9 spinal cord injury, T4–T5 compression fracture and T8–T9–T10 fracture)	PPSF (T6–T12) and a mini T9 vertebrectomy with T8–T10 decompression	No neurologic recovery (delayed treatment)	IV

**Table 3:** Literature on management of infectious spondylodiscitis using hybrid percutaneous pedicle screw fixation (PPSF).

Study	Type of Study	Number of Patients	Aim of Study/Spine Pathology	Type of Intervention	Outcomes	Level of Evidence (Oxford CEBM)
Kandwal et al. 2012 [30]	Retrospective analysis	38 (23 video thoracoscopic surgery (VATS), anterior debridement and fusion, 15 with PPSF and mini open and fusion)	To analyze outcomes from MISS; infections-especially tuberculous	VATS and anterior fusion vs. PPSF and mini-open debridement	Good fusion, less blood loss, better kyphosis correction	III
Garg and Vohra. 2014 [31]	Retrospective study	22 (posterior only treatment vs. anterior debridement and ventral column reconstruction)	To assess outcomes of MISS (extended vertebral body destruction); spine tuberculosis.	Transpendicular debridement and PPSF (vertebral body heights preserved) PPSF with ventral decompression and fusion (not preserved)	Neurological improvement, deformity control	III
Lin et al. 2014 [32]	Retrospective study	45 (20 of them with PPSF vs. open approach)	To compare MISS and open approach; pyogenic spondylodiscitis	Two stages procedure; Anterior debridement, fusion and PPSF vs. open posterior fixation	Less blood loss, better pain control, no recurrence	III
Lin et al. 2015 [33]	Retrospective study	22	To evaluate hybrid method (mini open anterior debridement with lumbar interbody fusion (ALIF) and PPSF; one level lumbar pyogenic spondylodiscitis	Mini-open anterior debridement and ALIF and PPSF	Safe, low complications, reduced tissue trauma	III
Wang et al. 2017 [34]	Retrospective study	22	To evaluate one stage procedure: extreme lateral channel interbody fusion (XLIF) and PPSF; lumbar spine tuberculosis	Debridement, fusion (XLIF) and PPSF	Shorter stay, faster recovery, less blood loss, infections and complications.	III
Zhang et al. 2020 [35]	Retrospective study	13 (11 with pyogenic spondylodiscitis and 2 with spine tuberculosis)	To present hybrid method: PPSF and mini-open approach; thoracolumbar spondylodiscitis	PPSF and mini open debridement and neural decompression	Less blood loss, surgical duration and better pain management	III

**Table 4:** Literature on metastatic spinal disease cases treated with hybrid spinal fixation techniques, including percutaneous pedicle screw fixation (PPSF).

Study	Type of Study	Number of Patients	Aim of Study/Spine Pathology	Type of Intervention	Outcomes	Level of Evidence (Oxford CEBM)
Lin et al. 2013 [36]	Retrospective study	25	To estimate long PPSF with decompression; metastatic disease	PPSF with open decompression (midline approach)	Safe, efficient, improves pain and neurological recovery	III
Rao et al. 2014 [37]	Retrospective study	8	To present a stratification system on use of MISS for metastatic spine disease	Stratified MISS: mini-open decompression and PPSF (short survival), mini-open vertebrectomy and PPSF (medium), open decompression and PPSF (long)	1/8 morbidity (wound infection), no perioperative mortality, operative duration and blood loss similar to other MISS	IV
Miscusi et al. 2015 [40]	Comparative study	42 patients (23 PPSF and minimally invasive laminotomy/laminectomy vs. 19 open procedure)	To compare MISS and open surgery; thoracic vertebral metastasis with myelopathy	PPSF and minimally invasive laminoto my/laminectomy vs. open decompression and instrumentation	Similar neurological recovery; MISS reduced length of stay, transfusions, opioid use	III
Hammad et al. 2017 [41]	Prospective study	51 (26 with PPSF and mini decompression)	To evaluate PPSF with or without mini decompression; symptomatic spinal metastasis	PPSF alone if no compression, PPSF and mini decompression if compression	Safe, maintains or improves functional outcome	III
Kumar et al. 2017 [38]	Prospective comparative study	45 (27 with MISS and 28 with open)	To compare results from MISS and open approaches; symptomatic metastatic spine disease	PPSF with midline microscopy-assisted decompression vs. open procedure	Comparable pain control, neurological and functional outcomes; earlier recovery	ΙII
Colangeli et al. 2020 [16]	Retrospective case series	52 (29 patients PPSF and mini spinal decompression and 23 PPSF only)	To assess MISS; spine metastasis	PPSF with/or without mini decompression	Similar neurological improvement and pain relief, fewer complications and shorter hospital stay	III

**Table 5:** Review articles on hybrid spinal fixation techniques, including percutaneous pedicle screw fixation (PPSF).

Study	Type of Study	Number of Patients	Aim of Study/Spine Pathology	Type of Intervention	Outcomes	Level of Evidence (Oxford CEBM)
Dhamija et al. 2021 [42]	Systematic review	31 studies	MISS and decompression in spinal metastasis	PPSF with mini decompression	Low complication rate and improved pain and neurological status	I
Luo et al. 2024 [43]	Narrative review article	-	To study treatment of with non-continuous multilevel spinal fractures	Hybrid open posterior fusion and PPSF	Early recovery, personalized approach	V
Sadh et al. 2026 [44]	Systematic review and meta-analysis	13 studies (912 patients, including 454 with open and 458 with percutaneous procedures)	To compare open vs. PPSF and lateral or anterior lumbar interbody fusion (LLIF or ALIF); spondylolysis, degenerative disk diseases or spondylolisthesis	PPSF and LLIF/ALIF vs. open posterior instrumentation	MISS better perioperative outcomes; open better deformity correction	I

**Table 6:** Summary of included studies on hybrid PPSF techniques according to pathology.

Pathology Group	Study	Study Design	Patients	Intervention	Main Outcomes
Degenerative/Spondylolisthesis	Lee et al., 2004 [17]	Retrospective	73	Mini-ALIF + PPSF	Less muscle injury, early discharge, good pain control
	Anderson et al., 2011 [28]	Retrospective	50	ALIF + PPSF	High fusion rate, significant pain improvement
	Kim et al., 2011 [39]	Retrospective	42	Mini decompression + multilevel PPSF	Less blood loss, improved outcomes
	Kotani et al., 2012 [45]	Prospective comparative	80	MIS-PLF + PPSF vs. open	Better function, lower complications
	Ulutaş et al., 2015 [47]	Prospective	70	TLIF through midline incision + PPSF	Good sagittal correction and absence of major complications
	Wang and Bordon, 2016 [48]	Retrospective	16	Pedicle subtraction osteotomy + PPSF	Reduced soft-tissue damage and satisfactory spinal alignment
	Barbagallo et al., 2014 [46]	Case series	13	TLIF + PPSF	Safe, no neurological deficits
	Heo et al., 2019 [49]	Comparative	65	PPSF + PLIF vs. open	Better alignment, improved outcomes
	Liu et al., 2020 [50]	Case–control	34	Osteotomy + PPSF	Less blood loss, similar correction
Trauma/Fractures/Kyphosis	Eck 2011 [18]	Case report	1	Lateral corpectomy + PPSF	Neurological improvement
	Sebastian et al., 2015 [19]	Case report	1	Hybrid long fusion + PPSF	Effective in complex trauma
	Kim et al., 2018 [20]	Case report	1	PPSF + mini decompression	Motion preservation, good outcome
	Park et al., 2018 [21]	Retrospective	27	PPSF + decompression	No neuro deterioration
	Ushijima et al., 2018 [22]	Case report	1	Open + percutaneous fixation	Successful multilevel fusion
	Erichsen et al., 2020 [23]	Retrospective	87	PPSF + anterior fusion	Less reduction loss, shorter surgery
	Todeschi et al., 2021 [25]	Prospective	110	PPSF ± staged fusion	Higher fusion rates
	Zhang et al., 2022 [26]	Comparative	64	PPSF + mini decompression	Less blood loss, good outcomes
	Bai et al., 2025 [27]	Retrospective	16	PPSF + endoscopic decompression	Good short-term outcomes
	Bravo et al., 2025 [29]	Case report	1	PPSF + vertebrectomy	Stabilization achieved
Infection (Spondylodiscitis/TB)	Kandwal et al., 2012 [30]	Retrospective	38	PPSF + debridement	Good fusion, less blood loss
	Garg and Vohra, 2014 [31]	Retrospective	22	PPSF + debridement	Neuro improvement
	Lin et al., 2014 [32]	Retrospective	45	ALIF + PPSF	Less pain, faster recovery
	Lin et al., 2015 [33]	Retrospective	22	Mini ALIF + PPSF	Safe, less complications
	Wang et al., 2017 [34]	Retrospective	22	XLIF + PPSF	Early recovery
	Zhang et al., 2020 [35]	Retrospective	13	PPSF + mini debridement	Less blood loss
Metastatic disease	Lin et al., 2013 [36]	Retrospective	25	PPSF + decompression	Pain relief, neuro recovery
	Rao et al., 2014 [37]	Retrospective	8	Stratified PPSF Approaches	Safe, low morbidity
	Miscusi et al., 2015 [40]	Comparative	42	PPSF + decompression	Less morbidity vs. open
	Hamad et al., 2017 [41]	Prospective	51	PPSF ± decompression	Safe, functional improvement
	Kumar et al., 2017 [38]	Prospective comparative	45	PPSF vs. open	Comparable outcomes
	Colangeli et al., 2020 [16]	Case series	52	PPSF ± decompression	Less complications
Review articles	Dhamija et al., 2021 [42]	Systematic review	31 studies	PPSF + decompression	Improved outcomes
	Luo et al., 2024 [43]	Narrative review	–	Hybrid fixation	Supports personalized surgery
	Sadh et al., 2026 [44]	Meta-analysis	912 patients	PPSF vs. open	MISS better perioperative outcomes

**Table 7:** Proposed pathology-stratified framework for selection of surgical approach.

Pathology	Full MIS	Hybrid PPSF	Open Surgery
Degenerative disease/spondylolisthesis	Isolated instability, limited fusion requirements	Need for decompression, interbody fusion, multilevel disease, moderate deformity correction	Severe deformity, major coronal/sagittal imbalance, complex revision surgery
Traumatic fractures	Stable fractures without neurological compression	Burst fractures with canal compromise, post-traumatic kyphosis, anterior column reconstruction	Highly unstable injuries, fracture-dislocations, extensive vertebral destruction
Infectious spondylodiscitis	Limited disease without instability	Debridement plus stabilization, neurological compression, moderate vertebral destruction	Extensive destruction requiring major reconstruction
Metastatic disease	Stabilization alone without significant compression	Mechanical instability with focal compression requiring limited decompression	Long survival expectancy, major vertebral body involvement, extensive tumor resection

## Data Availability

No new data were created or analyzed in this study. Data sharing is not applicable to this article.
